# Free-Living User Perspectives on Musculoskeletal Pain and Patient-Reported Mobility With Passive and Powered Prosthetic Ankle-Foot Components: A Pragmatic, Exploratory Cross-Sectional Study

**DOI:** 10.3389/fresc.2021.805151

**Published:** 2022-01-14

**Authors:** Andreas Kannenberg, Arri R. Morris, Karl D. Hibler

**Affiliations:** ^1^Department of Clinical Research and Services, Otto Bock Healthcare LP, Austin, TX, United States; ^2^Retired, Bradenton, FL, United States

**Keywords:** powered prosthetic ankle, powered prosthetic foot, powered prosthetic ankle-foot, knee pain, patient-reported mobility

## Abstract

**Introduction:**

Studies with a powered prosthetic ankle-foot (PwrAF) found a reduction in sound knee loading compared to passive feet. Therefore, the aim of the present study was to determine whether anecdotal reports on reduced musculoskeletal pain and improved patient-reported mobility were isolated occurrences or reflect a common experience in PwrAF users.

**Methods:**

Two hundred and fifty individuals with transtibial amputation (TTA) who had been fitted a PwrAF in the past were invited to an online survey on average sound knee, amputated side knee, and low-back pain assessed with numerical pain rating scales (NPRS), the PROMIS Pain Interference scale, and the PLUS-M for patient-reported mobility in the free-living environment. Subjects rated their current foot and recalled the ratings for their previous foot. Recalled scores were adjusted for recall bias by clinically meaningful amounts following published recommendations. Statistical comparisons were performed using Wilcoxon's signed rank test.

**Results:**

Forty-six subjects, all male, with unilateral TTA provided data suitable for analysis. Eighteen individuals (39%) were current PwrAF users, whereas 28 subjects (61%) had reverted to a passive foot. After adjustment for recall bias, current PwrAF users reported significantly less sound knee pain than they recalled for use of a passive foot (−0.5 NPRS, *p* = 0.036). Current PwrAF users who recalled sound knee pain ≥4 NPRS with a passive foot reported significant and clinically meaningful improvements in sound knee pain (−2.5 NPRS, *p* = 0.038) and amputated side knee pain (−3 NPRS, *p* = 0.042). Current PwrAF users also reported significant and clinically meaningful improvements in patient-reported mobility (+4.6 points PLUS-M, *p* = 0.016). Individuals who had abandoned the PwrAF did not recall any differences between the feet.

**Discussion:**

Current PwrAF users reported significant and clinically meaningful improvements in patient-reported prosthetic mobility as well as sound knee and amputated side knee pain compared to recalled mobility and pain with passive feet used previously. However, a substantial proportion of individuals who had been fitted such a foot in the past did not recall improvements and had reverted to passive feet. The identification of individuals with unilateral TTA who are likely to benefit from a PwrAF remains a clinical challenge and requires further research.

## Introduction

An amputation of a limb does not only remove passive anatomical structures but also results in the loss or truncation of muscles that are the actuators for movement and ambulation. Therefore, it appears consistent to develop powered prosthetic components that replace the function of the lost or impaired muscles. Thus far, however, passive components are still the standard of care in lower limb prosthetics. That requires individuals with amputations to adopt compensatory mechanisms to cope with the lack of power and active movement. In individuals with transtibial amputations (TTA), such compensations include slower walking speeds (1), about 25% higher energy expenditure for walking than able-bodied persons (2, 3), decreased sound limb step length (4), and reduced power generation in the residual knee (5). One important reason for these compensatory mechanisms is that passive prosthetic feet provide only up to 55% of the push-off power of the natural ankle-foot complex (6). Studies have shown that a commercially available powered prosthetic ankle-foot component (PwrAF) generates speed-dependent push-off power that may be comparable with that of the natural ankle (6–8). However, the results on its impact on function, such as self-selected walking speed (7, 9–13), metabolic energy expenditure on level ground (7, 9–11) and inclines (8, 10), patient-reported prosthetic function (12), and other aspects of prosthetic mobility have been inconclusive or conflicting.

Several studies have reported that walking with a PwrAF resulted in significant unloading of the knee joint of the sound limb (7, 13, 14). That is consistent with earlier findings that reduced push-off of the trailing limb requires increased collision work of the leading limb, which results in greater loading of its knee joint (15–17). This biomechanical evidence makes anecdotal reports from users of PwrAF on improved sound knee pain and pain-free walking distance noteworthy. Several studies with a PwrAF that did not find significant group benefits published the individual results of their subjects (9–11, 18). A thorough review of these subject-specific results revealed that, varying across the outcomes assessed, 35–50% of these individuals had experienced clinically meaningful benefits of the PwrAF during the studies. However, the published individual data has not allowed for narrowing down conclusive subject characteristics that would help guide the identification of individuals who are more likely to benefit from a PwrAF than others.

Therefore, it was decided to take a pragmatic, exploratory approach to systematically collect and analyze real-world, long-term user perspectives on musculoskeletal pain and prosthetic mobility in a bigger sample of individuals who were fitted a PwrAF in the past. The aim of the present study was to determine whether unsolicited anecdotal reports on reduced sound knee pain, amputated side knee pain, and low-back pain and as well as improved patient-reported prosthetic mobility were isolated occurrences or reflect a common experience in users of PwrAF. The results of the study, depending on the findings, were intended to serve as the basis for future planning of interventional studies.

## Methods

### Study Design and Procedure

This was a pragmatic, exploratory cross-sectional clinical practice study approved by the Institutional Review Board (IRB) of the Baylor College of Medicine, Houston, TX, USA. A total of 250 individuals who had been fitted a PwrAF were invited to participate. All potential subjects had given prior written permission to contact them through email for research projects. The invitation email contained a link to the survey administered through Qualtrics® survey software. Subjects first provided IRB-approved informed consent and then answered questions on their demographics. Subjects completed the outcome measures for their current foot and provided the recalled ratings for their previous prosthetic foot. All responses were anonymous and de-identified.

### The Device

**Figure 1:**
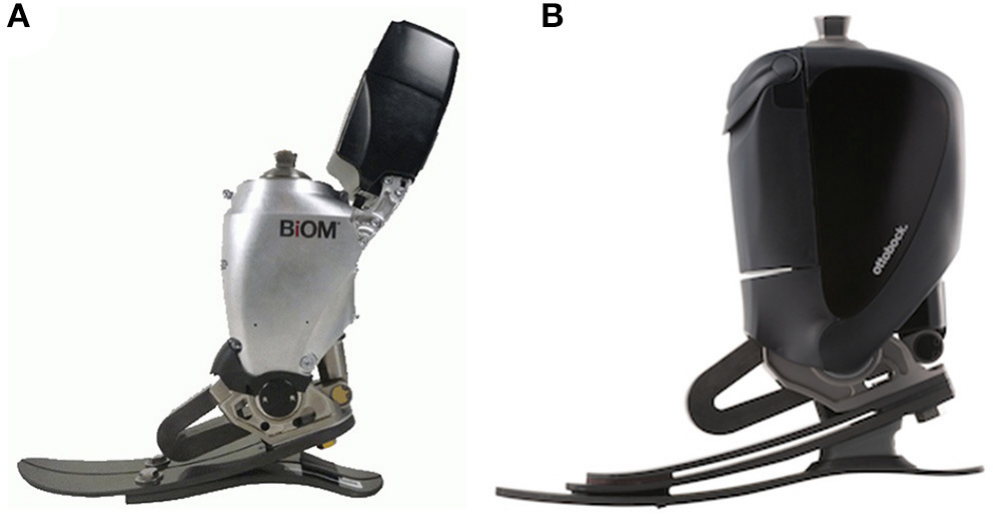
**(A)** BiOM® powered ankle-foot component. **(B)** Empower® powered ankle-foot component.

Subjects had been fitted with one of two versions of a PwrAF, either the BiOM® T2 (BionX, Bedford, MA, USA) or the Empower® (Ottobock Healthcare LP, Austin, TX, USA). The BiOM was the earlier version and had been marketed from 2012 to 2017, whereas the Empower is the current version that has been available since 2018. Both devices have a combined ankle-motor/U-spring actuator mounted on an energy-storage-and-return (ESR) foot platform (Figures 1A,B) to provide actively powered plantarflexion/push-off during gait. The amount of push-off power delivered depends on patient weight, walking speed, terrain, and tuning of the software and reaches the level of able-bodied individuals (6–8). Compared to the previous BiOM, the current Empower was designed to have a more compact design without the protruding battery arm, achieve more consistent power delivery by more efficient springs and improved tuning properties, and to extend battery life from a few hours to a full day.

### Survey

The survey form inquired demographic information (age in 20-year bins, gender, height, weight), details on the amputation level and etiology, socket style and design, as well as prosthetic components. Subjects also completed the following outcome measures: numerical pain rating scales (NPRS) for average sound knee, amputated side knee, and low-back pain, the PROMIS Pain Interference Form-6a, and the Prosthetic Limb Users' Survey of Mobility–PLUS-M^TM^ 12-item Short Form. The online survey form is provided as Supplementary Material.

### Outcome Measures

Numerical Pain Rating Scales (NPRS) are well-established and validated tools to assess pain on an 11-point scale from “0,” representing no pain, to “10” representing the most intense pain imaginable (19–21). Subjects were asked, “How much pain do you suffer on average using your current prosthetic foot?” and “How much pain did you suffer on average using your previous prosthetic foot?” Pain ratings from 1 to 3 are considered “mild,” 4 to 6 “moderate,” and 7 to 10 “severe” (22). The minimal clinically important difference (MCID) has been reported to be 1 point or a 15% change (20). Improvements of 2 points or 30% have been found to correspond with a “much better” verbal rating of patients (23, 24). Numerical pain rating scales have been validated and used for remote electronic data collection (25, 26).

The PROMIS Pain Interference item banks assess self-reported consequences of pain on relevant aspects of subjects' lives including to what extent pain hinders engagement with social, cognitive, emotional, physical, and recreational activities (27). The pain interference short forms are universal rather than disease specific. They have been validated for diverse populations (28–30). In this study, subjects completed the 6-item original short form 6a. The response format was a 5-point ordinal rating scale of “Not at all,” “A little bit,” “Somewhat,” “Quite a bit,” and “Very much.” Raw scores were converted to an item-response theory-based T-score using the PROMIS scoring manual (31). A T-score of 50 represents the average for the US general population with a standard deviation of 10 (27). A higher T-score indicates higher pain interference, and the MCID for T-score changes has been reported to be 2.0–3.0 (28, 29). The PROMIS Pain Interference has also been validated for remote (32) electronic data capture (30).

The PLUS-M is a validated and commonly used outcome tool based on a validated bank of 44 items to assess patient-reported mobility with a lower-limb prosthesis (33, 34). In this study, subjects completed the 12-item Mobility Short Form v1.1. The response format was a 5-point ordinal rating scale of “Unable to do,” “With much difficulty,” “With some difficulty,” “With a little difficulty,” and “Without any difficulty.” Raw scores were converted to T-scores using the validated conversion table in the PLUS-M User Guide (35). Higher T-scores indicate better self-reported mobility. The minimal detectable change (MDC) has been reported to be 4.5 points (36). The PLUS-M has also been validated for electronic data collection (36).

### Adjustment for Recall Bias

Patients are known to have a tendency to recall more pain but less functional limitations in retrospective postoperative assessments of preoperative knee and low-back pain and function compared to concurrent ratings prior to surgery (37–39). Therefore, an adjustment of the retrospective ratings was conducted to account for recall bias. Previous research comparing past concurrent and recalled ratings of pain and function reported an average recall error of 10% of the total range of the measurement tool (37). Thus, recalled pain ratings on the 0–10 NPRS were reduced by 1 point, except for original ratings of 0 or 1. For the PROMIS Pain Interference and the PLUS-M, the total raw scores for recalled ratings were reduced by 10% of their respective ranges, i.e., 2 points for the PROMIS Pain Interference (range 6–30) and 5 points for the PLUS-M (range 12–60), except if the raw score would have fallen below the minimum possible raw score. In that case, the raw score was adjusted to the minimum score. The recall-adjusted raw scores were then converted to T-scores as described above. However, adjustments for recall bias were not performed if they would have favored the PwrAF. Thus, recalled pain and pain interference ratings for the PwrAF in current passive foot (PAS) users as well as recalled PLUS-M ratings for PAS in current PwrAF users were not adjusted. We took this cautious methodological approach to reduce or possibly even prevent an overestimation of benefits of the PwrAF that subjects recalled and to prevent the creation of benefits that subjects did not recall.

### Statistical Analyses

Descriptive statistics for the ordinal variables include the median, interquartile ranges (IQR), minimum and maximum values, and for T-scores means and standard deviations. Differences between the PwrAF and PAS scores were evaluated using Wilcoxon's matched pairs signed ranks test due to substantial departures from the normal distribution. This non-parametric statistical test is appropriate for the analysis of data where measurements of the same individual respondent are obtained under different conditions. McNemar's chi-square was used to test the significance of differences in proportions between PWRAF and PAS. For all statistical tests, *p* < 0.05 were considered statistically significant.

Differences between PwrAF and PAS scores were analyzed for the two subject groups of current PwrAF users and current PAS users to avoid combining of current and recalled ratings for either foot in the statistical tests. In the group of current PwrAF users, current ratings for PwrAF were compared to the recalled original and adjusted ratings for PAS, whereas in the group of current PAS users, the current ratings for PAS were compared to the recalled original and adjusted ratings for PwrAF.

The individual results were analyzed in a descriptive way to find potential explanations for why each subject accepted or abandoned the PwrAF. Current and adjusted recalled ratings for the same outcome were analyzed for clinically meaningful differences between PwrAF and PAS. The three pain scores were summed up for a total pain score and differences ≥3 points NPRS were considered clinically meaningful. For the PROMIS Pain Interference and PLUS-M scores, differences ≥3.0 or ≥4.5 points were deemed clinically meaningful, respectively.

## Results

### Demographics

A total of 52 individuals answered all questions of the online survey. Three subjects with bilateral transtibial and three subjects with transfemoral amputation were excluded. The responses of 46 individuals with unilateral TTA, all male, were subjected to the data analysis. This dataset represents a response rate of 18.4%. The demographic details of the subjects are depicted in Table 1. There were no significant differences between the groups of current PwrAF and current PAS users.

**Table 1:** Demographics of the subjects.

	**Entire sample**	**Current PwrAF users**	**Current PAS users**
*N*	46	18	28
Sex male	46	18	28
Age			
20–39 years	8	2	6
40–59 years	23	9	9
60–79 years	14	6	8
80+ years	1	1	0
Height (cm)	181 ± 7	180 ± 6	182 ± 7
Weight (kg)	98.7 ± 15.4	100.2 ± 17.6	97.8 ± 13.7
Amputation etiology			
Trauma	37	16	21
Vascular disease	2	0	2
Cancer	1	1	0
Infection/Sepsis	4	1	3
Other	2	0	2
Time since amputation (years)	16.2 ± 11.3	19.1 ± 14.7	14.3 ± 7.8
Time of use of the PwrAF (years)	3.8 ± 3.0	6.6 ± 2.5	2.1 ± 1.8
Time of use of the PAS (years)	13.9 ± 10.9	16.0 ± 14.8	12.5 ± 6.9
Socket type with PwrAF			
Pin lock	14	4	10
Suction	17	9	8
Vacuum	11	4	7
Other	4	1	3
Socket type with PAS			
Pin lock	17	5	12
Suction	18	9	9
Vacuum	3	3	4
Other	1	1	3

Eighteen subjects (39%) identified as current PwrAF users. Twenty-eight subjects (61%) reported to have used a PwrAF in the past but abandoned it because of its weight, limited battery life, or lack of waterproofness.

### Musculoskeletal Pain

#### All Subjects

**Figure 2:**
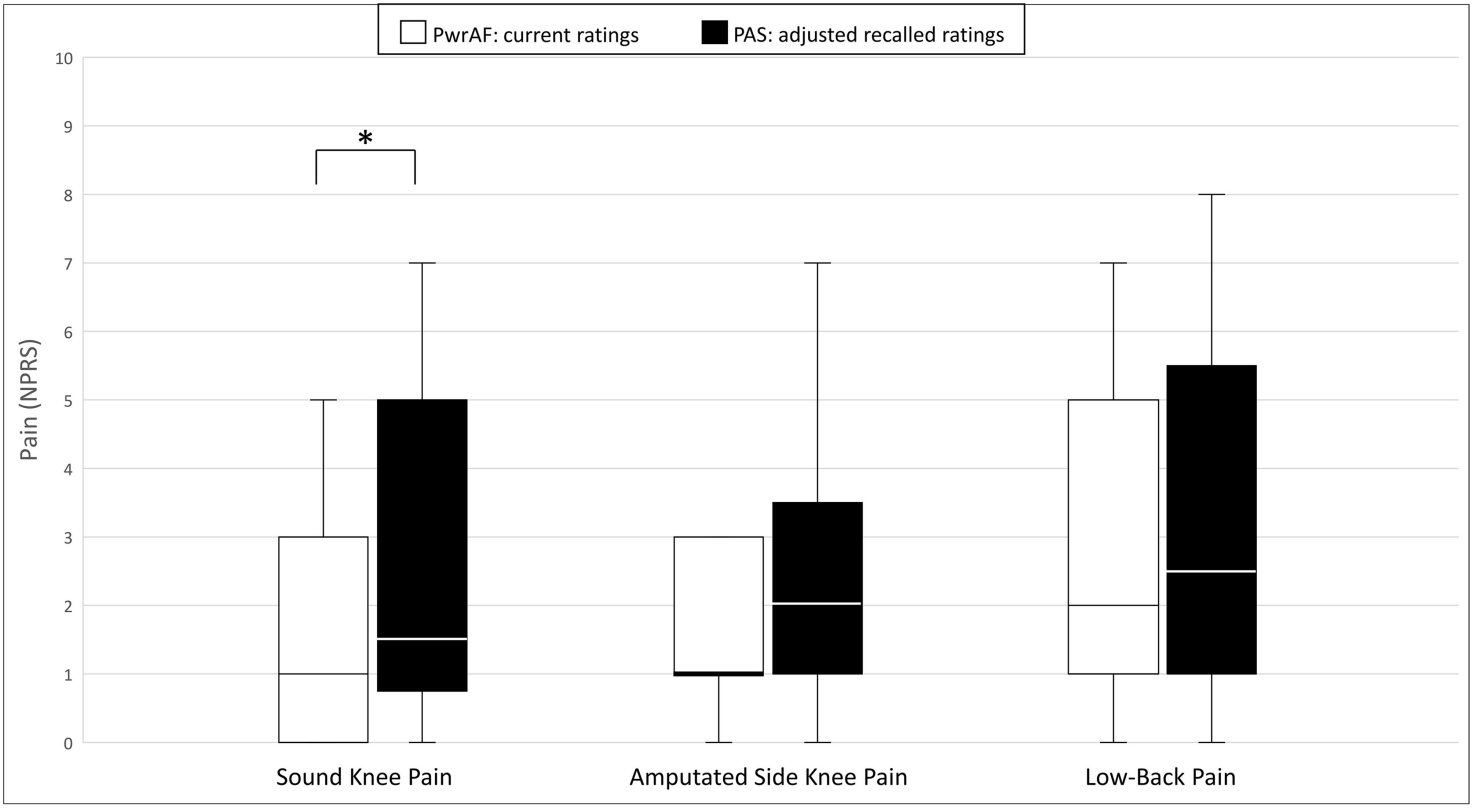
Sound knee pain, amputated side knee pain, and low-back pain in current PwrAF users (*n* = 18). *p < 0.05 (see text for details). Differences in medians of 1 point or greater are considered clinically meaningful.

In the original ratings, the 18 current PwrAF users reported significantly lower median current sound knee pain, amputated side knee pain, and low-back pain than they recalled for PAS. The difference in the PROMIS Pain Interference T-scores, though clinically meaningful in magnitude, did not reach statistical significance (Table 2). After adjustment of the recalled pain ratings for PAS for recall bias, only current sound knee pain remained significantly lower with PwrAF [1 (IQR 0–3) vs. 1.5 (IQR 0.75–5); *p* = 0.036] (Figure 2). The differences in amputated side knee pain [1 (IQR 1–3) vs. 2 (IQR 1–3.5); *p* = 0.12] and low-back pain [2 (IQR 1–5) vs. 2.5 (IQR 1–5.5); *p* = 0.33] were no longer statistically significant (Figure 2).

**Table 2:** Original pain and pain interference in current PwrAF users.

	**PwrAF** **current ratings**	**PAS** **recalled ratings**	***p*-value**
Sound knee pain [median (IQR)]	1 (0–3)	2.5 (0.75–6)	0.007
Amputated side knee pain [median (IQR)]	1 (1–3)	3 (1–4.5)	0.007
Low-back pain [median (IQR)]	2 (1–5)	3.5 (1.75–6.5)	0.011
PROMIS pain interference [mean (±SD)]	50.9 (±7.4)	53.8 (±10.0)	0.173

*IQR, interquartile range; SD, standard deviation*.

In the group of the 28 current PAS users, no statistically significant differences were seen between both foot types in the pain and pain interference ratings (Table 3).

**Table 3:** Original pain and pain interference in current PAS users.

	**PAS current ratings**	**PwrAF recalled ratings**	***p*-value**
Sound knee pain [median (IQR)]	1.5 (0–3.75)	1 (0–3)	0.131
Amputated side knee pain [median (IQR)]	1 (0–3.75)	1 (0–3.75)	0.473
Low-back pain [median (IQR)]	2 (1–4)	2 (0.25–4)	0.823
PROMIS Pain Interference [mean (±SD)]	53.2 (±10.1)	53.1 (±9.6)	0.965

*Recalled ratings for PwrAF were not adjusted (lowered) as this would have favored PwrAF. IQR, interquartile range; SD, standard deviation*.

#### Subjects Who Reported or Recalled Moderate to Severe Sound Knee Pain When Using PAS

After adjustment for recall bias, 13/46 subjects (28%; 6 PwrAF and 7 PAS users) reported current or recalled moderate to severe sound knee pain ≥4 NPRS with use of PAS, respectively. Most of these individuals also reported current or recalled amputated side knee and low-back pain at that level.

**Figure 3:**
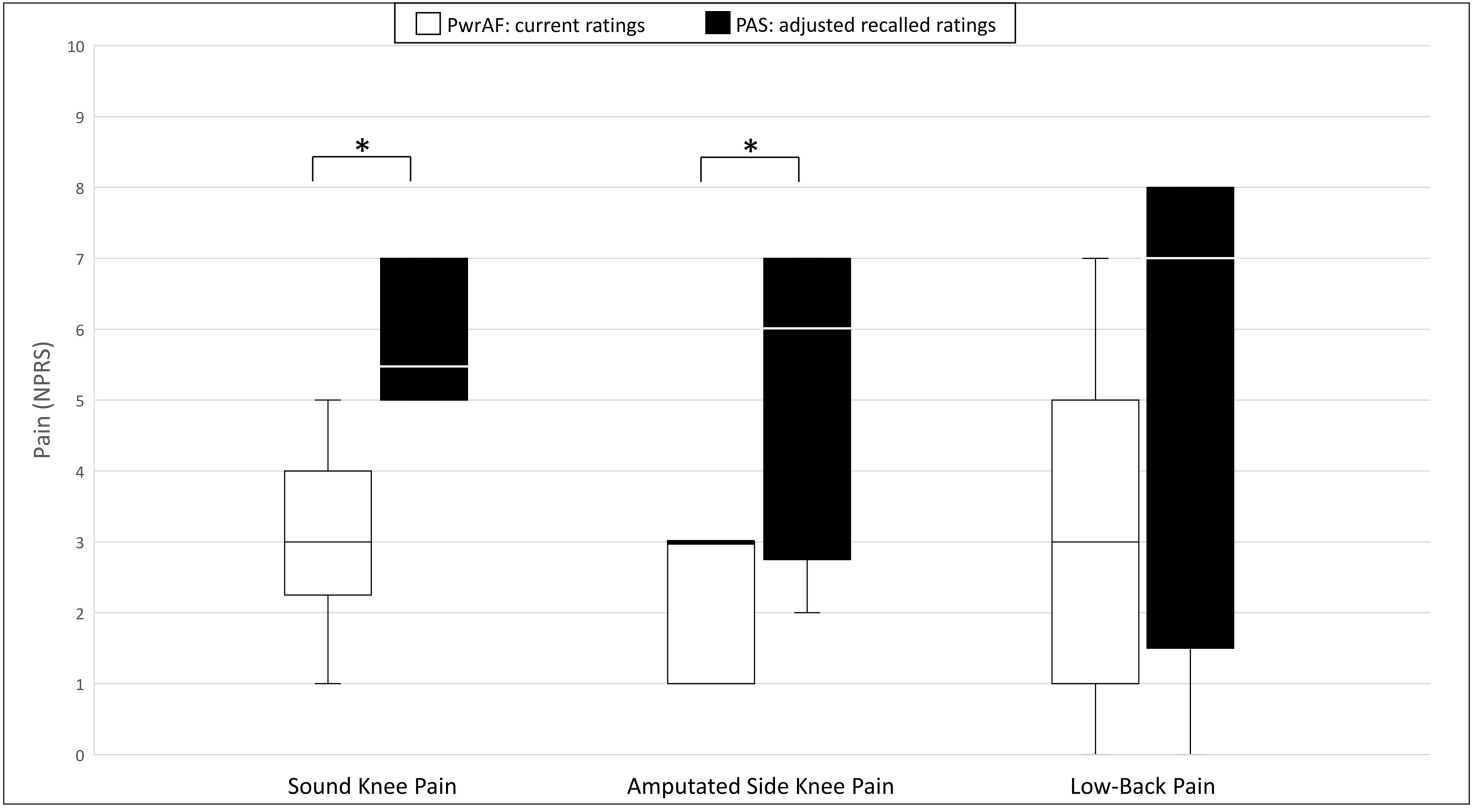
Sound knee pain, amputated side knee pain, and low-back pain in current PwrAF users (*n* = 18) who recalled moderate to severe sound knee pain ≥4 NPRS when using PAS. *p < 0.05 (see text for details). Differences in medians of 1 point or greater are considered clinically meaningful.

Current PwrAF users reported significantly and clinically meaningfully lower current median sound knee pain [3 (IQR 1.75–4.25) vs. 5.5 (IQR 5–7); *p* = 0.038] and amputated side knee pain [3 (IQR 1–3) vs. 6 (IQR 2.75–7), *p* = 0.042] than in the adjusted recalled ratings for their previous PAS. The differences in low-back pain [3 (IQR 0.75–5.6) vs. 7 (IQR 1.5–8); *p* = 0.068] and pain interference [54.5 ± 8.2 vs. 62.7 ± 4.2; *p* = 0.074), though clinically meaningful in magnitude, failed to attain statistical significance in this small subgroup (Figure 3).

In contrast, the current PAS users who reported current sound knee pain ≥4 NPRS did not recall any significant differences in pain and pain interference between the feet (Table 4).

**Table 4:** Pain and pain interference in current PAS users who reported sound knee pain ≥4 NPRS when using PAS.

	**PAS** **current ratings**	**PwrAF** **recalled ratings**	***p*-value**
Sound knee pain [median (IQR)]	5 (5–9)	5 (3–6)	0.063
Amputated side knee pain [median (IQR)]	4 (2–6)	4 (2–5)	0.465
Low-back pain [median (IQR)]	4 (3–7)	6 (2–7)	0.715
PROMIS Pain interference [mean (±SD)]	63.6 (±9.3)	61.9 (±8.5)	0.965

*Recalled ratings for PwrAF were not adjusted (lowered) as this would have favored PwrAF*.

*IQR, interquartile range; SD, standard deviation*.

### Patient-Reported Mobility

**Figure 4:**
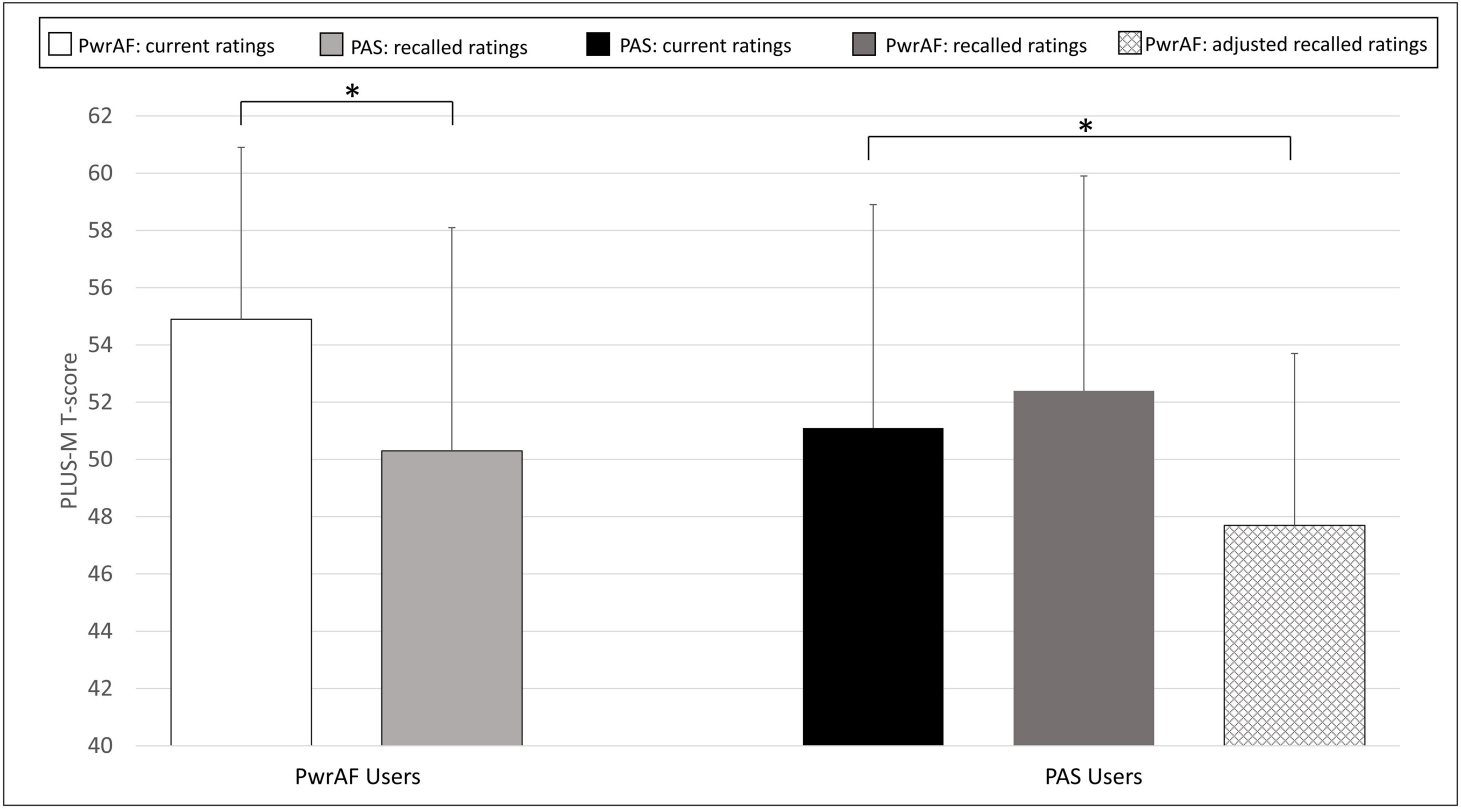
Mean PLUS-M T-scores in current PwrAF users (*n* = 18) and current PAS users (*n* = 28). *p < 0.05 (see text for details). Differences in means of 4.5 points or greater are considered clinically meaningful.

The group of 18 current PwrAF users reported significantly and clinically meaningfully higher current PLUS-M scores with PwrAF than they recalled for their previous PAS (54.9 ± 6.0 vs. 50.3 ± 7.8; *p* = 0.016) (Figure 4). No adjustment of the recalled PLUS-M ratings for PAS was performed as this would have further favored the PwrAF.

The 28 current PAS users did not recall a difference in PLUS-M scores with PwrAF to the current PLUS-M rating with PAS (52.4 ± 7.5 vs. 51.1 ± 7.8; p=0.071). The adjustment of the recalled PLUS-M ratings for PwrAF resulted even in a significant, though not clinically meaningful advantage for PAS (51.1 ± 7.8 vs. 47.4 ± 6.0; *p* = 0.001) (Figure 4).

**Figure 5:**
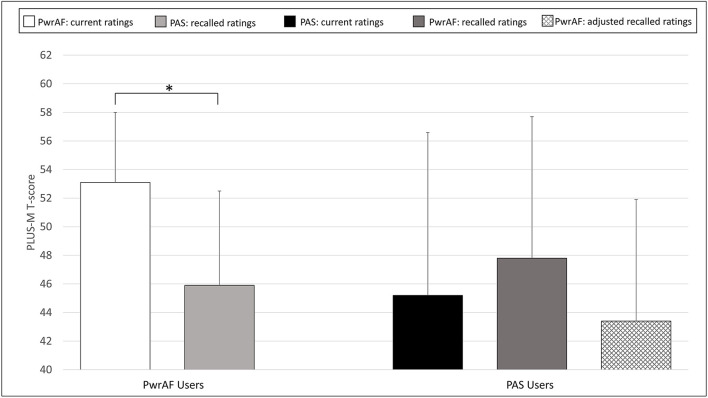
Mean PLUS-M T-scores in current PwrAF users (*n* = 6) and current PAS users (*n* = 7) who reported sound knee pain ≥4 NPRS when using PAS. *p < 0.05 (see text for details). Differences in means of 4.5 points or greater are considered clinically meaningful.

The 6 current PwrAF users with sound knee pain ≥4 NPRS in the adjusted recalled ratings for PAS reported significantly and clinically meaningfully higher current PLUS-M scores with PwrAF than they recalled for their previous PAS (52.8 ± 3.9 vs. 40.8 ± 4.6; *p* = 0.028). In contrast, the 7 current PAS users with sound knee ≥4 NPRS pain did not report a significant difference in mobility between the feet, not even after adjustment of the PLUS-M ratings for PwrAF for recall bias (PwrAF 43.4 ± 8.5 vs. PAS 45.2 ± 11.4; *p* = 0.735) (Figure 5).

### Group and Individual Outcomes by Version of the PwrAF

Of the 31 subjects who had been fitted the BiOM, only eight individuals (26%) were still using it at the time of the study. In contrast, 10 of the 15 individuals (67%) who had been fitted with an Empower were still current users.

The eight current BiOM users did not report any significant differences in pain, pain interference, and patient-reported mobility between PwrAF and PAS. After recall-adjustment, the 10 current Empower users reported significantly and clinically meaningfully lower current median sound knee pain [2.5 (IQR 1–3.25) vs. 4 (IQR 1–6.25); *p* = 0.043], amputated side knee pain [1 (IQR 1–3) vs. 2 (IQR 1–7); *p* = 0.041], and significantly and clinically meaningfully higher PLUS-M scores [55.1 ± 5.5 vs. 48.3 (±7.6); *p* = 0.012] than they recalled for the previous use of PAS.

On the individual level after adjustment for recall bias, 7 of the current 10 Empower users (70%) reported clinically meaningful improvements in the PLUS-M, and four subjects each (40%) in total pain and pain interference. Of the 8 current BiOM users, three individuals (37.5%) reported clinically meaningful improvements in the PLUS-M, and one subject each (12.5%) in total pain and pain interference. Summarizing the individual results, acceptance of the PwrAF may be explained by clinically meaningful improvements in the outcomes assessed in this study in 70% of current Empower and 37.5% of current BiOM users.

Likewise, abandonment of the PwrAF may be explained by the absence of clinically meaningful improvements in the outcomes used in this study in 5/5 (100%) former Empower and 17/23 (74%) former BIOM users. However, 6/23 individuals (26%) had abandoned the BiOM although they recalled clinically meaningful improvements when using the PwrAF.

## Discussion

The present pragmatic, exploratory study aimed at determining whether anecdotal reports of individuals with TTA on reduced musculoskeletal pain when using a PwrAF reflect a common experience among users or just isolated occurrences. As the identification of subjects who are likely to benefit from a PwrAF has been a clinical challenge (9–11), it was decided to survey a bigger sample of individuals who had been fitted such foot in the past to obtain real-world, long-term use experiences with the PwrAF and passive prosthetic feet. Though recall of ratings for previous interventions has limitations and challenges, this pragmatic approach is very similar to clinical practice where patients are usually asked to compare their current symptoms to those they recall for time points in the past. Recall of past symptoms and effects of sequential interventions is also the internal reference of the patient when a decision must to be made on the replacement of worn-out prosthetic components. However, as patients tend to overestimate past pain and function (37–39), recalled ratings were adjusted by clinically meaningful amounts according to recommendations in the literature (37), unless that would have favored the PwrAF. Therefore, it is reasonable to assume that statistically significant and/or clinically meaningful differences that still exist after such substantial adjustments are worth contemplating and discussing.

Due to the limitations of recall, the discussion will take a conservative approach and focus only on differences between the two types of prosthetic feet after adjustment for recall bias. Current PwrAF users reported significantly less sound knee pain than for use of a passive foot. If current PwrAF users had recalled moderate or even severe sound knee pain ≥4 NPRS when using a passive foot, they reported statistically significant and even greater, clinical meaningful improvements in sound knee pain and amputated side knee pain. They also reported clinically meaningful improvements in low-back pain and pain interference that, however, did not reach statistical significance. To the best of our knowledge, there are no published studies that have investigated the impact of specific prosthetic components on musculoskeletal pain. However, there are publications that confirm that musculoskeletal pain is a clinical problem in individuals with lower-limb amputations (40–42). Our findings on pain reduction are consistent with published biomechanical mechanisms for unloading the sound knee, residual knee, and lumbar region by increased push-off power and/or increased passive ankle range of motion in a prosthetic foot.

### Biomechanical Mechanisms for Unloading of the Sound Knee

For sound knee pain, it is important that push-off of the trailing limb produces forward and upward acceleration of the body's center of mass (COM) during step-to-step transition and reduces the collision work of the leading (sound) limb during landing (15–17). If the trailing prosthetic limb performs insufficient work during late stance phase to move the COM, the leading sound limb collides with the ground at a faster and downward directed speed (15), resulting in increased negative (eccentric) work to be performed by the sound limb's muscles and absorbed by its soft tissues and joints (17). Therefore, reduction of the negative work performed by the sound limb during collision may help reduce knee joint loading and the risk of developing knee osteoarthritis (43). The PwrAF investigated in this study has been shown to generate push-off that is comparable with that of able-bodied individuals (6–8). Consequently, biomechanical studies have found that the external knee adduction moment and other indicators of sound knee loading were reduced as compared to walking with a standard ESR foot (7, 13, 14). Though these reductions in knee loading were only statistically significant at faster walking speeds of 1.5 and 1.75 m/s (13, 14), they reached levels at medium walking speeds of 1.0 and 1.25 m/s that are considered effective for the treatment of knee osteoarthritis pain with knee unloader braces (44, 45). Thus, the reduction in sound knee pain found in this study may be explained by the biomechanical unloading of the knee of the sound limb associated with use of the PwrAF.

### Biomechanical Mechanisms for Unloading of the Amputated Side Knee

For the effect on amputated side knee pain, it is instrumental that the PwrAF mechanisms surveyed in this study have a plantarflexion range of motion of 22° that can be used passively for fast foot-flat during level walking and terrain adaptation. In contrast, most current prosthetic feet have no articulating components. It has been reported that prosthetic feet with a controlled ankle joint facilitate smoother rollover and faster progression of the center of pressure while diminishing or even eliminating the “dead spot” phenomenon that is caused by an inappropriate recoil of the heel spring at about 20% of stance phase. All these effects of a prosthetic ankle joint result in decreased loading of the residual knee (46). In addition, the adaptability of the prosthetic ankle on slopes and uneven terrain reduces biomechanical compensations on the prosthetic and sound limbs, facilitates faster foot-flat, improves the control of downhill walking speed, and significantly reduces the biomechanical loading of the residual knee (47–50). These effects that have been shown for passive feet with non-microprocessor and microprocessor-controlled ankles may also be assumed for the powered ankle-foot components surveyed and may explain the reduction in amputated side knee pain.

### Biomechanical Mechanisms for Unloading the Lumbar Spine and Muscles

For low-back pain, it is important that the loss of force and moment-generating capacity on the prosthetic side requires that the proximal muscles of the pelvis, hip, and lumbar spine participate in compensatory strategies to maintain balance and produce functional gait (51). These strategies often include complex recruitment of trunk muscles, co-activation of antagonistic muscles during stance, and asymmetric trunk posture at toe-off. While they support propulsion, they also result in high mechanical loads to the spine (51). Axial rotation of the lumbar spine is also increased during double-limb support, which may be a consequence of asymmetric trunk muscle strength and recruitment between the two legs (52, 53). These kinematic alterations in individuals with lower-limb amputation result in larger loads, loading rates, and load shifts compared to able-bodied individuals and are important risk factors that contribute to the onset of low-back pain (51). Increased prosthetic push-off has been shown to allow for better gait propulsion and force dissipation along the kinetic chain, thus reducing mechanical forces on proximal joints such as the knee, hip, and lumbar vertebrae (51, 54). As the prosthetic push-off produced by the powered ankles surveyed in this study reaches the natural push-off of able-bodied individuals (6–8), it may reduce asymmetries in pelvic and trunk muscle activation and improve force dissipation about the lumbar spine, thus alleviating low-back pain.

### Current PwrAF Users Reported Increased Prosthetic Mobility

In addition to the reduction in musculoskeletal pain, current PwrAF users also reported a statistically significant and clinically meaningful increase in patient-reported mobility. This improvement was also significant and even twice as big in subjects who had recalled moderate to severe sound knee pain when using a passive foot. Only one earlier study had investigated patient-reported mobility with a PwrAF and did not find significant differences to passive feet in their sample (12). Possible explanations for the improvement in patient-reported mobility with PwrAF use are the support of propulsion and ambulation by powered push-off and, in subjects with sound knee pain with PAS use, a reduction in musculoskeletal pain.

### Why Don't All Individuals With TTA Benefit From a PwrAF?

As impressive as the results for pain reduction and patient-reported mobility in current users of a PwrAF are, it needs to be highlighted that a substantial proportion of individuals in our study had reverted to a passive foot and did not recall any differences during the time when they had used a PwrAF. On the individual level, even among the current PwrAF users, only 55% of subjects reported clinically meaningful benefits in patient-reported mobility and/or pain. Our findings are consistent with other studies that found either no or only limited benefits of a PwrAF in their entire samples, but detailed results showed that about 35–50% of their subjects had benefitted individually in walking speed, metabolic energy consumption, daily activity, or other aspects of prosthetic mobility (9–11).

That raises the question why only some persons with TTA appear to benefit from using a PwrAF. A systematic review of studies on prosthetic push-off power found that powered push-off had its greatest effects at walking speeds of ≥1.22 m/s, suggesting that individuals with high physical capabilities might be more likely to benefit from a PwrAF (6, 9). However, a later study found that 40–50% of its subjects who walked at slower speeds were also able to benefit from the PwrAF in self-selected in-lab walking speed and/or cost of transport (11). Another plausible explanation has been suggested by Kim et al. who studied muscle activation patterns when using a PwrAF (55). In able-bodied persons, push-off and propulsion are mainly driven by the gastrocnemius muscle (53), whereas current PwrAF act like the soleus muscle (10, 55) that has its main function in standing and postural control (56). As the external power is transferred differently than in able-bodied individuals (to the socket and residual limb below the knee vs. to the femur) and not integrated in the neuromuscular control of the user, subjects with TTA have to alter their neuromuscular control strategy to react to the added power and utilize it for propulsion (57). However, the study of Kim et al. did not find consistent muscle activation patterns in subjects with TTA while walking with a PwrAF, not even in long-term users (52). That suggests that motor learning and adaptation of neuromuscular control to the PwrAF may not be intuitive. Without a dedicated training program, some individuals with good motor learning skills may learn it fast, some may need a longer time, but a substantial portion of subjects may never learn to master it on their own. This is supported by our finding and that of others that only about half of the subjects benefitted individually and a few individuals even recalled more pain when using the PwrAF. Therefore, the current evidence suggests that the development of a specific gait training and rehabilitation program may help increase the proportion of individuals with TTA who could benefit from a PwrAF. For example, increasing the activity of the residual limb rectoris femoris muscle during walking may be a good strategy to stabilize the residual knee against flexion and, as a result, utilize the external ankle power more effectively by facilitating its transfer to the femur. In addition, activity of the gluteus medius and the medial hamstring on the amputated side appear to be correlated with the metabolic cost for walking (55).

### Differences Between the Current and Previous Versions of the PwrAF

There were notable differences between the current Empower and the previous BiOM versions of the PwrAF. The proportion of current users was much higher for the Empower (67%) than for the BiOM (26%), and current Empower users reported significantly higher patient-reported mobility as well as significantly less sound knee and amputated side knee pain than with use of passive feet, while current BiOM users did not. Individuals who had abandoned either version reported no differences in the outcomes between PwrAF and passive feet.

On the individual level, acceptance of the Empower could be explained by clinically meaningful improvements in 70% of current users, while this was the case in only 37.5% of current BiOM users. Interestingly, more subjects experienced clinically meaningful improvements in patient-reported mobility than in pain. Abandonment of either PwrAF could be explained by the absence of individual clinically meaningful benefits in 79% of subjects. However, 21% of passive foot users, all former BiOM users, abandoned the PwrAF although they had recalled clinically meaningful benefits with its use. The likely reason for that is that the drawbacks of the BiOM technology, such as higher weight and limited battery life, outweighed the benefits for these individuals. Previous studies with a PwrAF only investigated short-term benefits and preference of the technology and did not report long-term benefits or device acceptance (7–14, 58).

There may be three possible explanations for the differences in clinical benefits and acceptance between the Empower and the BiOM. First, users of the Empower have been exposed to the technology for a much shorter period than users of the BiOM. Benefits may wear off or become less important and drawbacks more bothersome over time, especially as individuals age and decline in physical capacity. Second, technological improvements in the Empower in tuning, springs, and consistency of power delivery may have improved the ease of adapting neuromuscular control compared to the BiOM. Third, it cannot be ruled out that the sample of current Empower users consisted, by chance, of a greater number of individuals with excellent motor learning skills who had mastered the adaptation of their neuromuscular control and were therefore able to utilize the external power effectively. Further research is needed to identify patient characteristics and factors in the technology and rehabilitation program that help increase the number of responders who benefit from PwrAF.

## Limitations

This study has limitations. First, it used recall for pain and prosthetic mobility for prosthetic feet that subjects had used in the past and compared them to ratings for the currently used prosthetic foot. Subjects are known to tend to overrate past pain and physical function as compared to current ratings taken in the past (37–39). The risk of recall bias in this study was addressed by adjusting the recalled ratings by clinically meaningful amounts following recommendations in the literature (37). In addition, these recall adjustments were only performed if they resulted in a disadvantage for the PwrAF by narrowing the differences to the passive feet. A second limitation is the current inability to define predictive characteristics of responders to the PwrAF. Thus, our study could only survey a sample whose majority had not benefitted from using the PwrAF. A third limitation of this study is that no information was available on other factors that may have had an impact on musculoskeletal pain associated with prosthesis use, such as prosthetic alignment or concurrent medical treatments, such as physical therapy. Future research should assess musculoskeletal pain prospectively with current ratings of the studied devices and consider potential confounding factors. Third, of the 250 potential subjects asked to participate in the survey, only 52 (20.8%) responded, all of them male and 80% with traumatic amputations. It is unknown whether the results are representative for the entire population and may also be transferable to female individuals, subjects with other amputation etiologies, or whether the sample was overly skewed toward individuals who did not benefit from the PwrAF.

## Conclusions

Free-living current users of powered prosthetic ankle-foot components reported significant and clinically meaningful improvements in patient-reported prosthetic mobility as well as sound knee and amputated side knee pain compared to recalled mobility and pain with passive feet used previously. However, a substantial proportion of individuals who had been fitted such a foot in the past did not recall improvements and had reverted to the use of passive feet. The rates of long-term acceptance and clinically meaningful benefits of the PwrAF device were much higher with the current than with the previous version. The identification of individuals with unilateral TTA who are likely to benefit from a PwrAF remains a clinical challenge and requires further research efforts. Nevertheless, a PwrAF is an option in the arsenal of the prosthetist and may be considered for individuals with unilateral TTA who suffer from musculoskeletal pain while using a passive prosthetic foot.

## Data Availability Statement

The raw data supporting the conclusions of this article will be made available by the authors, without undue reservation.

## Ethics Statement

The studies involving human participants were reviewed and approved by Institutional Review Board of the Baylor College of Medicine, Houston, TX, USA. The patients/participants provided their written informed consent to participate in this study.

## Author Contributions

AK had the study idea, contributed to the study design, data analysis, and wrote the sections Introduction and Discussion. AM contributed to the study design, developed the survey, performed data collection, contributed to the data analysis, and wrote the sections Methods and Results. KH performed the statistical analyses. All authors contributed to the article and approved the submitted version.

## Funding

This study was funded by a research grant of Otto Bock Healthcare LP, Austin, TX, USA.

## Conflict of Interest

AK and AM are full-time employees of Otto Bock Healthcare LP, the manufacturer of the product studied. Otto Bock provided the list of 250 individuals who were fitted with the product in the past and had given written permission to contact them for research projects. Otto Bock Healthcare had no influence on the study design, data analysis, and interpretation of the data. The remaining author declares that the research was conducted in the absence of any commercial or financial relationships that could be construed as a potential conflict of interest.

## Publisher's Note

All claims expressed in this article are solely those of the authors and do not necessarily represent those of their affiliated organizations, or those of the publisher, the editors and the reviewers. Any product that may be evaluated in this article, or claim that may be made by its manufacturer, is not guaranteed or endorsed by the publisher.

## Supplementary Material

Supplementary Data Sheet 1 Online survey form.
