# Supplementary material for: Free-Living User Perspectives on Musculoskeletal Pain and Patient-Reported Mobility With Passive and Powered Prosthetic Ankle-Foot Components: A Pragmatic, Exploratory Cross-Sectional Study
Source: Front Rehabil Sci. 2022 Jan 14;2:805151. doi: 10.3389/fresc.2021.805151 (PMC9397861; doi:10.3389/fresc.2021.805151)
Supplement: Supplementary Data Sheet 1 — Online survey form. [file Data_Sheet_1.pdf]

Powered Prosthetic Ankle Survey

Start of Block: Default Question Block

- I confirm that I have read and understood the Participant Information Sheet for the above project and the researcher has answered any queries to my satisfaction.
- I confirm that I have read and understood the Privacy Notice for Participants in Research Projects and understand how my personal information will be used and what will happen to it (i.e. how it will be stored and for how long).
- I understand that my participation is voluntary and that I am free to withdraw from the project at any time, up to the point of completion, without having to give a reason and without any consequences. To withdraw, I can close this window at any time.
- I understand that no protected health information is being collected for this survey.
- I understand that anonymised data (i.e. data that do not identify me personally) cannot be withdrawn once they have been included in the study.
- I understand that any information recorded in the research will remain confidential and no information that identifies me will be made publicly available.
- I consent to being a participant in the project.

○ I agree (1)

Page Break

Q1 Do you currently have an Powered ankle prosthesis and a non-powered prosthesis?

- Yes (1)
- No (2)

Q2 How old are you?

- Less than 20 years old
- 20 – 39
- 40 – 59
- 60 – 79
- 80 years or older

Q3 What is your gender?

- Male
- Female

Q4 What is your height and weight?

- Height (ft.) (1a) ____
- Height (in.) (1b) ____
- Weight (lbs) (2) ____

Q5 What is your level of amputation?

Right:	None (N/A)	Below the Knee (TT)	Above or at the Knee (KD/TF)	At the hip (HD)
Left:	None (N/A)	Below the Knee (TT)	Above or at the Knee (KD/TF)	At the hip (HD)

Q6 How many years has it been since your amputation?

Right: ________________________________

Left: ________________________________

Q7 What was the cause of your amputation?

____

Q8 On an average day, which of the following best describes your physical ability, regardless of the COVID pandemic?

○ I cannot walk without assistance from another person, even with a prosthetic (K0)

○ I can only get around the house without assistance (K1)

○ I can go outside, but do not get far without assistance (K2)

○ I can get around the community (e.g., grocery store or park) without assistance, but I am not particularly athletic (K3)

○ I run, jog, or otherwise do strenuous activities such as sports or heavy lifting (K4)

Q9 What is/was your socket suspension method for your prosthesis with the powered ankle prosthesis?

○ Pin (1)

○ Lanyard (2)

○ Suction (3)

○ Vacuum (4)

○ Other: (5) ____

Q10 What is/was your socket suspension method for your prosthesis with the non-powered ankle prosthesis?

○ Pin (1)

○ Lanyard (2)

○ Suction (3)

○ Vacuum (4)

○ Other: (5) ____

Q11 How long have you worn/been wearing (in months) your prosthesis with the:

○ Powered ankle prosthesis (1)
____

○ Non-powered ankle prosthesis (2)
____

Q12 What type of foot do/did you use with the powered ankle prosthesis?

○ Manufacturer: (1) ____

○ Model: (2) ____

Q13 What type of foot do/did you use with the previous non-powered prosthesis?

○ Manufacturer: (1) ____

○ Model: (2) ____

Q14 Did you need to use any medications for pain when wearing your prostheses?
Check all that apply (note that it is not possible to answer "yes" AND "no")

With the powered ankle prosthesis	With your non-powered prosthesis
○ Yes, a prescription strength pain medication	○ Yes, a prescription strength pain medication
○ Yes, an over-the counter pain medication	○ Yes, an over-the counter pain medication
○ I took medication for something other than pain	○ I took medication for something other than pain
○ No, I did not require any medication	○ No, I did not require any medication

Page Break

Q15 On a scale of 0-10 with 0 being the most uncomfortable socket and 10 being the most comfortable socket imaginable, how comfortable is your socket?

	0 1 2 3 4 5 6 7 8 9 10
(1)	

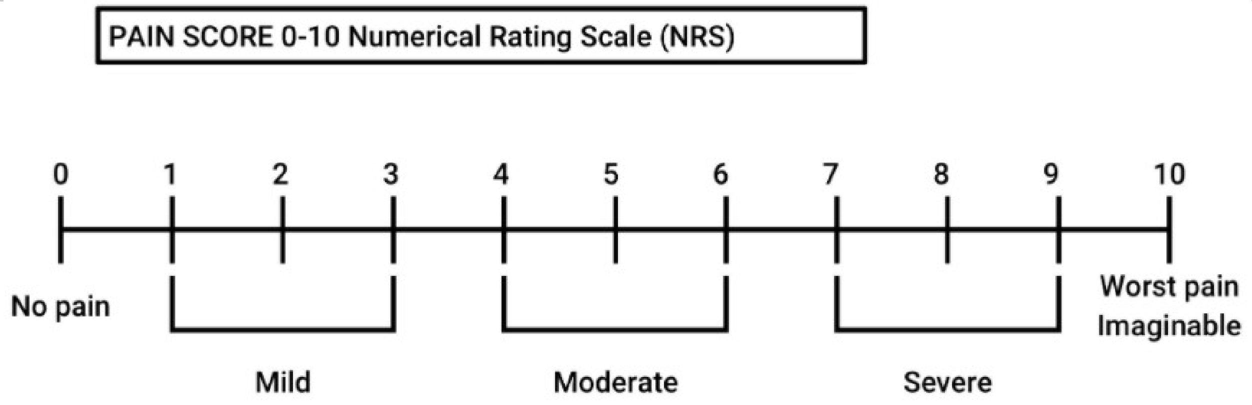

Q16 On a Scale of 0-10 with 0 being no pain at all and 10 being the worst pain imaginable, how much ***sound side knee pain*** do/did you experience daily when walking on your prosthesis:

	0 1 2 3 4 5 6 7 8 9 10
Powered ankle prosthesis (1)	
Non-powered prosthesis (2)	

Q17 On a Scale of 0-10 with 0 being no pain at all and 10 being the worst pain imaginable, how much ***amputated side knee pain*** do/did you experience daily when walking on your prosthesis:

	0 1 2 3 4 5 6 7 8 9 10
Powered ankle prosthesis (1)	
Non-powered prosthesis (2)	

Q18 On a Scale of 0-10 with 0 being no pain at all and 10 being the worst pain imaginable, how much ***back pain*** do/did you experience daily when walking on your prosthesis:

	0 1 2 3 4 5 6 7 8 9 10
Powered ankle prosthesis (1)	
Non-powered prosthesis (2)	

Q19 Which prosthesis do you use currently on a daily basis?

○ Powered ankle prosthesis (1)

○ Non-powered prosthesis (2)

○ None (3) Explain how you move: ________________________________

Page Break

In this section we would like to learn more about how pain interferes with an average week in your everyday life. For your current prosthesis, consider the past 7 days…

Q20 How much did pain interfere with your enjoyment of life?

With the Powered ankle prosthesis	With your non-powered prosthesis
○ Not at all (1)	○ Not at all (1)
○ A little bit (2)	○ A little bit (2)
○ Somewhat (3)	○ Somewhat (3)
○ Quite a bit (4)	○ Quite a bit (4)
○ Very much (5)	○ Very much (5)

Q21 How much did pain interfere with your ability to concentrate?

With the powered ankle prosthesis	With your non-powered prosthesis
○ Not at all (1)	○ Not at all (1)
○ A little bit (2)	○ A little bit (2)
○ Somewhat (3)	○ Somewhat (3)
○ Quite a bit (4)	○ Quite a bit (4)
○ Very much (5)	○ Very much (5)

Q22 How much did pain interfere with your day to day activities?

With the powered ankle prosthesis	With your non-powered prosthesis
○ Not at all (1)	○ Not at all (1)
○ A little bit (2)	○ A little bit (2)
○ Somewhat (3)	○ Somewhat (3)
○ Quite a bit (4)	○ Quite a bit (4)
○ Very much (5)	○ Very much (5)

Q23 How much did pain interfere with your enjoyment of recreational activities?

With the powered ankle prosthesis	With your non-powered prosthesis
○ Not at all (1)	○ Not at all (1)
○ A little bit (2)	○ A little bit (2)
○ Somewhat (3)	○ Somewhat (3)
○ Quite a bit (4)	○ Quite a bit (4)
○ Very much (5)	○ Very much (5)

Q24 How much did pain interfere with doing your tasks away from home (e.g., getting groceries, running errands)?

With the powered ankle prosthesis	With your non-powered prosthesis
○ Not at all (1)	○ Not at all (1)
○ A little bit (2)	○ A little bit (2)
○ Somewhat (3)	○ Somewhat (3)
○ Quite a bit (4)	○ Quite a bit (4)
○ Very much (5)	○ Very much (5)

Q25 How often did pain keep you from socializing with others?

With the powered ankle prosthesis	With your non-powered prosthesis
○ Not at all (1)	○ Not at all (1)
○ A little bit (2)	○ A little bit (2)
○ Somewhat (3)	○ Somewhat (3)
○ Quite a bit (4)	○ Quite a bit (4)
○ Very much (5)	○ Very much (5)

Page Break

In this section we would like to learn more about your function in everyday life with your prosthesis. Please respond to all questions as if you were wearing the prosthetic leg(s) specified. If you would normally use a cane, crutch, or walker to perform the task, please answer the questions as if you were using that device.

Q26 Are you able to walk a short distance in your home?

With the powered ankle prosthesis	With your non-powered prosthesis
○ Without any difficulty (5)	○ Without any difficulty (5)
○ With a little difficulty (4)	○ With a little difficulty (4)
○ With some difficulty (3)	○ With some difficulty (3)
○ With much difficulty (2)	○ With much difficulty (2)
○ Unable to do (1)	○ Unable to do (1)

Q27 Are you able to step up and down curbs?

With the powered ankle prosthesis	With your non-powered prosthesis
○ Without any difficulty (5)	○ Without any difficulty (5)
○ With a little difficulty (4)	○ With a little difficulty (4)
○ With some difficulty (3)	○ With some difficulty (3)
○ With much difficulty (2)	○ With much difficulty (2)
○ Unable to do (1)	○ Unable to do (1)

Q28 Are you able to walk across a parking lot?

With the powered ankle prosthesis	With your non-powered prosthesis
○ Without any difficulty (5)	○ Without any difficulty (5)
○ With a little difficulty (4)	○ With a little difficulty (4)
○ With some difficulty (3)	○ With some difficulty (3)
○ With much difficulty (2)	○ With much difficulty (2)
○ Unable to do (1)	○ Unable to do (1)

Q29 Are you able to walk over gravel surfaces?

With the powered ankle prosthesis	With your non-powered prosthesis
○ Without any difficulty (5)	○ Without any difficulty (5)
○ With a little difficulty (4)	○ With a little difficulty (4)
○ With some difficulty (3)	○ With some difficulty (3)
○ With much difficulty (2)	○ With much difficulty (2)
○ Unable to do (1)	○ Unable to do (1)

Q30 Are you able to move a chair from one room to another?

With the powered ankle prosthesis	With your non-powered prosthesis
○ Without any difficulty (5)	○ Without any difficulty (5)
○ With a little difficulty (4)	○ With a little difficulty (4)
○ With some difficulty (3)	○ With some difficulty (3)
○ With much difficulty (2)	○ With much difficulty (2)
○ Unable to do (1)	○ Unable to do (1)

Q31 Are you able to walk while carrying a shopping basket in one hand?

With the powered ankle prosthesis	With your non-powered prosthesis
○ Without any difficulty (5)	○ Without any difficulty (5)
○ With a little difficulty (4)	○ With a little difficulty (4)
○ With some difficulty (3)	○ With some difficulty (3)
○ With much difficulty (2)	○ With much difficulty (2)
○ Unable to do (1)	○ Unable to do (1)

Q32 Are you able to keep walking when people bump into you?

With the powered ankle prosthesis	With your non-powered prosthesis
○ Without any difficulty (5)	○ Without any difficulty (5)
○ With a little difficulty (4)	○ With a little difficulty (4)
○ With some difficulty (3)	○ With some difficulty (3)
○ With much difficulty (2)	○ With much difficulty (2)
○ Unable to do (1)	○ Unable to do (1)

Q33 Are you able to walk on an unlit street or sidewalk?

With the powered ankle prosthesis	With your non-powered prosthesis
○ Without any difficulty (5)	○ Without any difficulty (5)
○ With a little difficulty (4)	○ With a little difficulty (4)
○ With some difficulty (3)	○ With some difficulty (3)
○ With much difficulty (2)	○ With much difficulty (2)
○ Unable to do (1)	○ Unable to do (1)

Q34 Are you able to keep up with others when walking?

With the powered ankle prosthesis	With your non-powered prosthesis
○ Without any difficulty (5)	○ Without any difficulty (5)
○ With a little difficulty (4)	○ With a little difficulty (4)
○ With some difficulty (3)	○ With some difficulty (3)
○ With much difficulty (2)	○ With much difficulty (2)
○ Unable to do (1)	○ Unable to do (1)

Q35 Are you able to walk across a slippery floor?

With the powered ankle prosthesis	With your non-powered prosthesis
○ Without any difficulty (5)	○ Without any difficulty (5)
○ With a little difficulty (4)	○ With a little difficulty (4)
○ With some difficulty (3)	○ With some difficulty (3)
○ With much difficulty (2)	○ With much difficulty (2)
○ Unable to do (1)	○ Unable to do (1)

Q36 Are you able to walk down a steep gravel driveway?

With the powered ankle prosthesis	With your non-powered prosthesis
○ Without any difficulty (5)	○ Without any difficulty (5)
○ With a little difficulty (4)	○ With a little difficulty (4)
○ With some difficulty (3)	○ With some difficulty (3)
○ With much difficulty (2)	○ With much difficulty (2)
○ Unable to do (1)	○ Unable to do (1)

Q37 Are you able to hike about 2 miles on uneven surfaces, including hills?

With the powered ankle prosthesis	With your non-powered prosthesis
O Without any difficulty (5)	O Without any difficulty (5)
O With a little difficulty (4)	O With a little difficulty (4)
O With some difficulty (3)	O With some difficulty (3)
O With much difficulty (2)	O With much difficulty (2)
O Unable to do (1)	O Unable to do (1)

End of Block: Default Question Block

Thank you for participating in the Powered Prosthetic Ankle Survey! If you have felt uncomfortable with regards to your amputation, we recommend reaching out to a local support group through the Amputee Coalition by using the following link: https://www.amputee-coalition.org/support-groups-peer-support/how-to-find-support/
